# Personal Networks and Mortality Risk in Older Adults: A Twenty-Year Longitudinal Study

**DOI:** 10.1371/journal.pone.0116731

**Published:** 2015-03-03

**Authors:** Lea Ellwardt, Theo van Tilburg, Marja Aartsen, Rafael Wittek, Nardi Steverink

**Affiliations:** 1 University of Cologne, Cologne Graduate School in Management, Economics and Social Sciences (CGS), Cologne, Germany; 2 Vrije Universiteit Amsterdam, Department of Sociology, Amsterdam, The Netherlands; 3 University of Groningen, Department of Sociology and Interuniversity Center for Social Science Theory and Methodology (ICS), Groningen, The Netherlands; 4 University Medical Center Groningen, University of Groningen, Department of Health Psychology, Groningen, The Netherlands; Carnegie Mellon University, UNITED STATES

## Abstract

**Background:**

Research on aging has consistently demonstrated an increased chance of survival for older adults who are integrated into rich networks of social relationships. Theoretical explanations state that personal networks offer indirect psychosocial and direct physiological pathways. We investigate whether effects on and pathways to mortality risk differ between functional and structural characteristics of the personal network. The objective is to inquire which personal network characteristics are the best predictors of mortality risk after adjustment for mental, cognitive and physical health.

**Methods and Findings:**

Empirical tests were carried out by combining official register information on mortality with data from the Longitudinal Aging Study Amsterdam (LASA). The sample included 2,911 Dutch respondents aged 54 to 85 at baseline in 1992 and six follow-ups covering a time span of twenty years. Four functional characteristics (emotional and social loneliness, emotional and instrumental support) and four structural characteristics (living arrangement, contact frequency, number of contacts, number of social roles) of the personal network as well as mental, cognitive and physical health were assessed at all LASA follow-ups. Statistical analyses comprised of Cox proportional hazard regression models. Findings suggest differential effects of personal network characteristics on survival, with only small gender differences. Mortality risk was initially reduced by functional characteristics, but disappeared after full adjustment for the various health variables. Mortality risk was lowest for older adults embedded in large (HR = 0.986, 95% CI 0.979—0.994) and diverse networks (HR = 0.948, 95% CI 0.917—0.981), and this effect continued to show in the fully adjusted models.

**Conclusions:**

Functional characteristics (i.e. emotional and social loneliness) are indirectly associated with a reduction in mortality risk, while structural characteristics (i.e. number of contacts and number of social roles) have direct protective effects. More research is needed to understand the causal mechanisms underlying these relations.

## Introduction

A vast body of research in social epidemiology has established a substantial impact of social integration on health and survival [1–3]. There is mounting evidence that large and diverse personal networks reduce the risk of common diseases in older adults, including elevated blood pressure and cardiovascular dysfunction [4], ischemic heart disease [5], cancer [6], cognitive impairment [7] and dementia [8]. Socially well supported adults are also more likely to recover from severe illnesses, such as breast cancer [9] and myocardial infarction [5], and ultimately live longer than older adults with inadequate social relationships [2].

Several pathways have been proposed to explain the health effects of personal networks, two of which are mostly used [10]. First, networks provide psychological and material resources intended to benefit an individual’s ability to effectively cope with stress during adverse events, thereby indirectly promoting health [11]. The perceived quality of social relationships, that is the availability of emotional and instrumental support and the absence of loneliness, concern *functional characteristics* of the personal network. Second, social integration offers opportunities for participation in a broad range of relationships together with a sense of communality and identification with one’s social roles [10]. Personal networks are a source of fulfillment of basic human attachment needs, positive psychological states and social pressure to take care of oneself, all of which directly—and independently of the former stress-buffering effects—induce health-promoting physiological responses [3, 11, 12]. Quantitative aspects of social relationships, most importantly number and diversity of an individual’s contacts (i.e. with a partner, friends, relatives, colleagues or neighbors), denote *structural characteristics* of the personal network. Based on these arguments, the question arises whether and how social integration reduces the risk of mortality.

Evidence from previous research on mortality is inconclusive in two respects. First, findings are inconsistent with regard to which network characteristics are the best predictors of mortality risk. Some studies found stronger benefits of functional characteristics [13, 14], others found structural characteristics [15, 16] as the major source of life prolonging effects. Whereas Holt-Lunstad *et al*. [2] summarize and break down findings across multiple studies by structural and functional measures in their meta-analytic review, the current study examines the relative and independent effects of both measures within a single sample.

Second, the role of change in personal networks has been addressed insufficiently. Previous work has shown that personal networks may undergo drastic modifications in later life, e.g. they typically shrink and change in composition [17, 18], also because older adults become selective in their relationship investments when they see their time horizon shrinking [19]. Yet, most studies have predicted mortality based on a sole baseline measurement rather than following personal networks over time. As a result, time spans between network predictors and mortality outcome have varied much across studies: Holt-Lunstad *et al*. [2] recorded follow-up time spans ranging from three months to 58 years, with an average lag of 7.5 years. Predicting mortality in the distal future (i.e. applying a long time lag) likely yields biased results and limits the comparability of studies. In sum, findings are strongly determined by choice of personal network characteristics and time lags.

The objective of the present study is to examine the association between mortality risk and both functional and structural network characteristics, after adjustment for mental, cognitive and physical health, and accounting for changes in the personal network. Empirical tests are carried out by combining official register information on mortality with data from the Longitudinal Aging Study Amsterdam (LASA), which assessed personal networks of older adults for twenty years.

## Methods

### Study population

LASA is an ongoing longitudinal, multidisciplinary research project focusing on physical, emotional, cognitive and social functioning in later life. The LASA sample is a nationally representative sample of older adults aged 55–85 years at baseline. Participants were recruited from municipal registries within three geographic regions in the Netherlands, with an oversampling of older individuals and older men in particular. Since 1992, data have been collected every three years using the same face-to-face interviews and self-administered questionnaires. The data collection was approved by the Committee on the Ethics of Research in Humans of the Faculty of Medicine at VU University Amsterdam. As part of the baseline interview, respondents were asked to fill in an informed consent form, stating that they have been adequately informed about their participation in LASA and that they agree to participate.

For an observation to be selected into the analysis, a respondent had to have complete information on all variables under study (i.e. no missing values) for the time point of this observation. The analysis included 2,911 participants in total, using the first LASA observation (1992–1993) and six follow-up observations in 1995–1996, 1998–1999, 2001–2002, 2005–2006, 2008–2009 and 2011–2012. Table 1 shows the number of participants in the different follow-up periods. The 1,413 men and 1,498 women were followed for a maximum of 20 years (*M* = 9.1; *SD* = 5.7). On average, 3.5 valid observations were available for each respondent, summing to a total of 10,031 observations.

**Table 1:** Life-table of participants.

Period			Number			Survival	
Start	End	Eligible ^a^	Included	Deaths	Lost to follow-up	Rate	95% CI
1992/3	1995/6	3,069	2,911	350	317	0.873	0.860–0.885
1995/6	1998/9	2,537	2,244	256	249	0.767	0.751–0.783
1998/9	2001/2	2,039	1,739	184	194	0.681	0.662–0.700
2001/2	2005/6	1,650	1,361	202	198	0.572	0.551–0.593
2005/6	2008/9	1,226	961	101	114	0.508	0.486–0.530
2008/9	2011/2	960	746	100	119	0.434	0.411–0.457
2011/2	1–11–2013	598	527	28	n/a	0.391	0.365–0.416

*Notes*.

^a^ Confirmed eligible when information on vital status was available.

### Measurements

#### Mortality

Participants’ vital status was retrieved up to 1 November 2013 through linkage with population register data. Duration of survival was calculated in days and rescaled into years for graphical interpretations. Our defined period of observation started on the date of a participant’s first interview and ended five years after the date of a participant’s last interview. This five-year cut-off was chosen to ensure that predictors remained proximate to the timing of the outcome. Although periods between observations were designed to last approximately three years, they may have lasted four to five years, particularly when multiple attempts were needed to establish contact and interview participants. We therefore opted for a cut-off value longer than one regular period but shorter than two periods. However, observation stopped no later than the register data’s endpoint of 1 November 2013. In case of death during an observation period, days of survival were counted between the first interview date and the decease date. In case of no death during observation, days of survival were counted between the first interview date and the end date of the observation period. Death hazard was predicted based on four functional and four structural personal network characteristics that have typically been used in previous research [2]. These eight predictors are specified below.

#### Functional predictors

Feelings of *emotional* and *social loneliness* were measured with the two-dimensional 11-item De Jong Gierveld Loneliness Scale [20]. Social loneliness relates to missing a wider social network, while emotional loneliness refers to missing an intimate relationship. This distinction implies that respondents may report relatively rich social lives but feel lonely nevertheless. There are six statements on social loneliness, e.g. “there is always someone I can talk to about my day-to-day problems”, and five statements on emotional loneliness, e.g. “I experience a general sense of emptiness”. Possible answers are “yes”, “more or less”, and “no”. Scores for positively formulated items were reversed. Answers were dichotomized, so that “yes” and “more or less” indicate loneliness (1) versus “no” loneliness (0). Scores were summed, such that high scores indicated severe loneliness. A separate personal network module asked questions on the participants’ set of social relationships [21]. Participants were first asked to identify people with whom they had regular and important socially active contacts. For the nine most frequent contacts—other than the partner—it was asked how much support participants had received: For *emotional support* one question was asked “How often in the past year did you talk to [name] about your personal experiences and feelings?”. For *instrumental support* one item assessed “How often in the past year did [name] help you with daily tasks in and around the house?”. Responses ranged from “1 = never” to “4 = often”, and were averaged across all answers for each support type.

#### Structural predictors

For their living arrangement it was assessed whether participants *lived alone* (1 = yes) or with a partner (0 = no). *Contact frequency* was measured within the above-mentioned personal network module, using the question “How often are you in touch with [name]?”. Possible responses ranged from “1 = never” to “8 = daily”, and were averaged across all answers. *Network size* was obtained through counting all identified contacts in the personal network. *Network diversity* was assessed with a slightly adapted version of the Cohen’s Social Network Index [22]. This was the number of social roles in which a respondent had regular—i.e. biweekly or more often—contact with at least one person. Contacts were classified into 13 distinct social roles: spouse, child, child-in-law, sibling, sibling-in-law, parent, relative, close friend, acquaintance, neighbor, (former) colleague, voluntary organization, other group member. Respondents received one point for every role covered by their regular contacts.

#### Mental health

Self-report scales informed on participants’ mental health. First, the 20-item CES-D scale assessed *depressive symptoms* experienced within the past week [23]. Second, *anxiety* over the past four weeks was captured with seven items from the HADS scale [24]. Items were summed for each scale, with higher values indicating stronger symptomatology.

#### Cognitive health

Cognitive functioning was measured with the Mini-Mental State Examination (*MMSE*), a widely used 23-item screening instrument [25]. This index covers several dimensions of cognition, such as recall, orientation, registration, attention, language, and construction. Higher values indicated better cognitive functioning.

**Physical health.** Two measures captured physical health. First, the capacity to carry out *activities of daily living* (ADL) was determined with six questions [26]. A sum score was computed, so that high scores indicated good physical functioning. Second, the total *number of chronic diseases*, i.e. of lung, heart, arteries, diabetes, CVA (stroke), arthritis and cancer, was used in the analysis.

### Analytical strategy

Using Cox proportional hazard regressions, we predicted the outcome of interest, mortality, based on the participants’ network characteristics. Participants’ network and health variables were repeatedly assessed—up to seven times—and thus incorporated as time-varying covariates. Table 2 summarizes all variables under study at baseline, and Table 3 presents the intercorrelations among the four functional and four structural predictor variables. All Cox models controlled for age at baseline. The analysis was stratified for gender, as women had a much higher survival rate than men (*χ* ^2^(1) = 90.34, *p*<0.001) and differed in many variables. Stratification allows the baseline death hazards to differ by group (i.e. strata), while the parameter coefficients are constrained to be the same. However, we also fitted models for men and women separately to obtain separate coefficients. This was to compare the predictors’ relations with mortality between men and women. Supporting information with a full overview of the results for the complete set of variables is available in the Tables I to XXVII in the S1 File. Here we solely report the hazard ratios (HRs) for the predictor variables. This is because our analytical strategy produced many models.

**Table 2:** Characteristics of male and female study participants at baseline ^a^.

Characteristic	All participants		Men		Women		*t*-test	
	*M*	*SD*	*M*	*SD*	*M*	*SD*	*| t |*	*p*
*Control variable*								
Age in years	70.36	8.69	70.54	8.70	70.19	8.67	1.09	0.27
*Functional predictors*								
Emotional loneliness	1.13	1.66	0.92	1.48	1.33	1.80	6.76	0.00
Social loneliness	0.92	1.33	0.99	1.36	0.86	1.31	2.76	0.01
Emotional support	1.71	0.77	1.59	0.80	1.82	0.72	8.40	0.00
Instrumental support	0.80	0.73	0.82	0.74	0.78	0.71	1.67	0.09
*Structural predictors*								
Living alone	0.35	—	0.21	—	0.49	—	233.35 ^b^	0.00
Contact frequency	5.69	0.91	5.68	0.97	5.68	0.91	0.16	0.87
Network size	13.86	8.25	13.76	8.33	13.95	8.17	0.63	0.53
Network diversity	4.52	1.84	4.43	1.83	4.60	1.85	2.55	0.01
*Mental health*								
Depression	7.74	7.59	6.40	6.58	9.01	8.25	9.39	0.00
Anxiety	2.57	3.32	2.06	2.87	3.04	3.64	8.00	0.00
*Cognitive health*								
MMSE	27.06	2.69	27.07	2.66	27.05	2.72	0.25	0.80
*Physical health*								
No. of chronic diseases	0.64	0.88	0.69	0.88	0.60	0.87	2.08	0.01
ADL	27.36	4.53	28.11	3.68	26.65	5.10	8.80	0.00
*N*	2,911		1,413		1,498			

*Notes*.

^a^ The baseline measurement concerned a participant’s first complete observation, i.e. without missing values.

^b^ For the dichotomous variable *living alone* the gender difference was tested with a χ^2^-test (df = 1).

**Table 3:** Intercorrelations among the eight predictors at baseline ^a^.

	Emotional loneliness	Social loneliness	Emotional support	Instrumental support	Living alone	Contact frequency	Network size
*Functional predictors*							
Emotional loneliness	—						
Social loneliness	0.40***	—					
Emotional support	−0.09***	−0.20***	—				
Instrumental support	0.04*	−0.09***	0.17***	—			
*Structural predictors*							
Living alone	0.39***	0.15***	−0.02	0.10***	—		
Contact frequency	−0.05**	−0.10***	0.08***	0.20***	−0.06**	—	
Network size	−0.20***	−0.31***	0.12***	0.06***	−0.19***	−0.31***	—
Network diversity	−0.23***	−0.35***	0.15***	0.06***	−0.31***	0.14***	0.61***

*Note*.

^a^ *N* = 2,911.

The baseline measurement concerned a participant’s first complete observation, i.e. without missing values.

*p < 0.05

**p < 0.01

***p < 0.001

The following steps were undertaken to test the predictors’ associations with mortality. First, in a series of eight baseline models, the main effects of the eight predictors were estimated separately, that is per model only one predictor was included (together with the control variable age). These Models 1 informed on the age-adjusted effect of each predictor on mortality risk. Second, all eight predictors were tested jointly in an extended Model 2. To avoid multicollinearity, we proceeded with the separate baseline models. In following steps, these baseline models were adjusted for mental, cognitive and physical health respectively (Models 3–5). In Model 6 the baseline models were adjusted for all of the former health variables. Finally, the longitudinal Model 7 tested whether a network characteristic became more or less influential on mortality risk as time had passed. For this analysis, an interaction effect predictor × time (specified with the tvc-option in *Stata 13*.*1* software) was added to the previous model. Table 4 provides an overview of the modeling strategy.

**Table 4:** Modeling steps of the Cox proportional hazard regressions.

Models	Description	Variables
1	Baseline model ^a^	Age + single predictor
2	Extended model	Age + single predictor + remaining predictors
3	Mental health	Age + single predictor + mental health
4	Cognitive health	Age + single predictor + cognitive health
5	Physical health	Age + single predictor + physical health
6	Total health ^b^	Age + single predictor + mental health + cognitive health + physical health
7	Total health and time ^c^	Age + single predictor + mental health + cognitive health + physical health + interaction single predictor × time

*Notes*. All models were stratified for gender.

^a^ This is the age-adjusted model.

^b^ This is the fully adjusted model.

^c^ Results of Model 7 are not reported in the Cox regression table but in the text only.

## Results

### Age-adjusted baseline models

Table 5 presents the results from the Cox models. Older adults who felt emotionally or socially lonely and received much instrumental support exhibited increased mortality risks (Models 1). Furthermore, mortality risk was lower for older adults living with their partner, reporting many contacts and great diversity in their personal network, compared to older adults with small and less diverse networks. Neither frequency of contact, nor emotional support were associated with mortality. Model 2 largely resembled the findings from the age-adjusted baseline Models 1, except that the positive effects of social loneliness and living alone on mortality had disappeared.

**Table 5:** Death hazard ratios from Cox proportional hazard models for different predictors, with adjustment for potential confounders (*N*_ind_ = 2,911, *N*_obs_ = 10,031).

	**Models 1**	**Model 2**	**Models 3**	**Models 4**	**Models 5**	**Models 6**
		Baseline model adjusted for ^a^				
	Baseline model	Extended model	Mental health	Cognitive health	Physical health	Total health
**Predictor**	HR (95% CI)	HR (95% CI)	HR (95% CI)	HR (95% CI)	HR (95% CI)	HR (95% CI)
*Functional predictors*						
Emotional	1.079***	1.053**	1.020	1.073***	1.039*	1.023
loneliness	(1.047,1.113)	(1.017,1.090)	(0.985,1.055)	(1.041,1.106)	(1.007,1.071)	(0.989,1.059)
Social	1.067***	1.016	1.028	1.060**	1.046*	1.030
loneliness	(1.028,1.108)	(0.973,1.062)	(0.989,1.068)	(1.022,1.101)	(1.008,1.087)	(0.991,1.070)
Emotional	0.985	0.975	0.989	1.003	0.994	1.009
support	(0.917,1.058)	(0.904,1.051)	(0.921,1.062)	(0.934,1.077)	(0.926,1.067)	(0.940,1.083)
Instrumental	1.137**	1.154***	1.102*	1.127**	1.056	1.049
support	(1.053,1.227)	(1.063,1.252)	(1.021,1.190)	(1.045,1.216)	(0.978,1.141)	(0.971,1.132)
*Structural predictors*						
Living	1.230**	1.066	1.107	1.203**	1.097	1.051
alone	(1.082,1.399)	(0.929,1.223)	(0.971,1.262)	(1.058,1.368)	(0.963,1.251)	(0.920,1.199)
Contact	1.075*	1.039	1.077*	1.052	1.060	1.048
frequency	(1.012,1.143)	(0.969,1.113)	(1.014,1.143)	(0.991,1.117)	(0.998,1.126)	(0.988,1.112)
Network	0.978***	0.988*	0.982***	0.983***	0.982***	0.986***
size	(0.970,0.986)	(0.977,0.999)	(0.975,0.990)	(0.975,0.990)	(0.974,0.990)	(0.979,0.994)
Network	0.919***	0.957	0.937***	0.934***	0.932***	0.948**
diversity	(0.889,0.950)	(0.913,1.003)	(0.906,0.968)	(0.904,0.966)	(0.901,0.963)	(0.917,0.981)

*Notes*. 95% confidence intervals in brackets.

*p < 0.05

**p < 0.01

***p < 0.001.

All models controlled for age at baseline and were stratified by gender.

^a^ Models 1 tested the age-adjusted effect of a single predictor. Model 2 adjusted for the remaining predictors, thus testing the total set of predictor variables in a joint model. Models 3 adjusted for depression and anxiety (mental health). Models 4 adjusted for the MMSE-index (cognitive health). Models 5 adjusted for number of chronic diseases and ADL (physical health). Models 6 adjusted for all mental, cognitive and physical health variables.

### Functional predictors adjusted

Neither emotional, nor social loneliness were associated with mortality, once mental health was added to the model (Models 3). This suggests that pathways from loneliness to mortality operate through mental disorders: Older adults who reported feelings of loneliness showed stronger symptoms of anxiety (*r*_emotional_ = 0.37, *p*<0.001; *r*_social_ = 0.18, *p*<0.001) and depression (*r*_emotional_ = 0.49, *p*<0.001; *r*_social_ = 0.25, *p*<0.001), which in turn lowered their chance of survival. Receipt of instrumental support continued to elevate mortality risk, but this was explained by physical impairments (Models 5): Highly supported individuals had slightly more chronic diseases (*r* = 0.10, *p*<0.001) and poorer physical functioning (*r* = −0.19, *p*<0.001) than less supported individuals. Notably, none of the functional predictors were associated with mortality after full adjustment with the complete set of health variables (Models 6).

### Structural predictors adjusted

**Fig 1:**
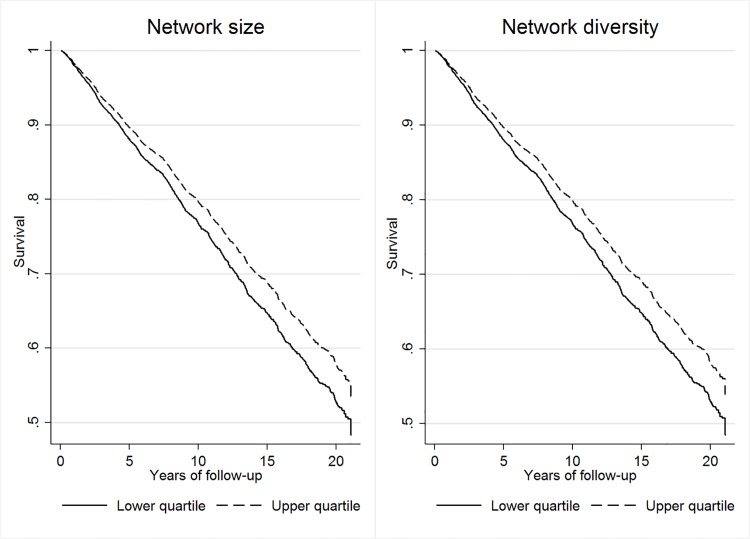
Survivor functions compared for upper and lower quartiles of network structure (*N*_ind_ = 2,911). *Note*. Based on predictions from the fully adjusted Cox regression models (Models 6). For network size, the lower quartile (25th percentile) included 8 contacts, while the higher quartile (75th percentile) included 19 contacts. For network diversity, the lower quartile included 3 social roles, while the higher quartile included 6 social roles.

The rather large baseline effect of living alone on mortality did not show after adjustment for mental (Models 3) and physical health (Models 5). Risk of mortality did not vary with contact frequency once cognitive (Models 4) and physical health (Models 5) were included in the model. These characteristics were thus indirectly related to mortality and multiple paths were possible. In contrast, the remaining structural characteristics were directly associated with mortality: Older adults embedded in large and diverse personal networks had lower risks of mortality in all adjusted models, even after adding the full set of health variables (Models 6). One additional contact in the network yielded a risk reduction of about 2%, and one additional social role implied a reduction of 5% in death hazard within five years after the last network measurement. Fig. 1 illustrates the differences in mortality risk for integration into poor versus rich network structures, i.e. risk in the highest relative to the lowest quartile.

### Sensitivity analyses

#### Gender differences

**Fig 2:**
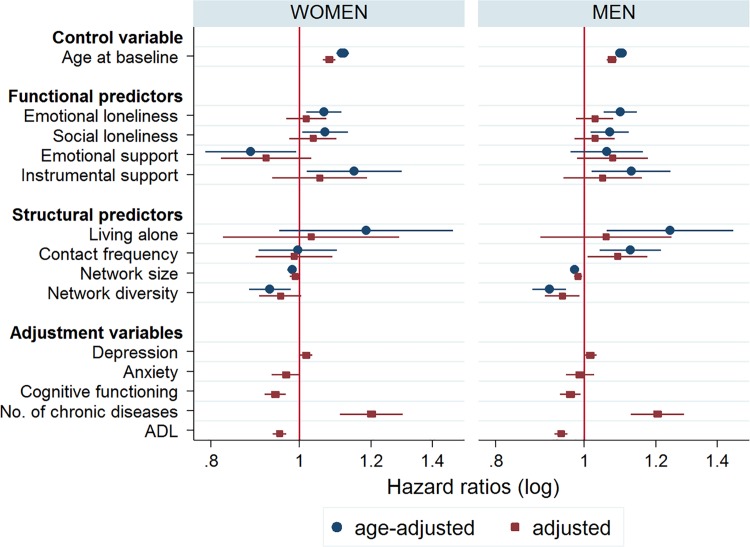
Death hazard ratios for women (*N*_ind_ = 1,498) and men (*N*_ind_ = 1,413), compared for age-adjusted and adjusted models. *Note*. Hazard ratios are shown on a logarithmic scale. Age-adjusted coefficients represent bivariate associations from Models 1. Adjusted coefficients represent multivariate associations from Models 6. Hazard ratios may not be compared across the different variables (as ranges are unequal), but only between age-adjusted and adjusted coefficients, and between men and women.

To see whether the predictors’ influences were sensitive to gender differences, we re-ran the models separately for men and women. Fig. 2 compares the resulting hazard ratios and their corresponding 95% confidence intervals from the age-adjusted (Models 1) and the fully adjusted models (Models 6). Point estimates graphed towards the left indicate reduced hazards, while estimates towards the right hint at escalated risk of mortality. There is no significant association with mortality when the confidence interval crosses the horizontal line of HR = 1. Not surprisingly, all of the previously age-adjusted point estimates for the personal network characteristics shifted closer to this line after full adjustment. The many characteristics of the personal network had similar associations with mortality in both men and women, with few exceptions. Men had somewhat higher death hazards than women when living alone and having frequent contacts, but decreased risk in large and diverse networks. Women experienced greater chance of survival than men when surrounded by emotionally supportive contacts. Note that these gender differences were not statistically significant after full adjustment.

#### Time-varying associations

To test whether associations of mortality with network characteristics became stronger or weaker towards the end of the life-span, we added an interaction variable with time to the fully adjusted models (Models 7, not reported). The results yielded no significant interaction estimates for any of the eight variables, suggesting that associations with personal networks do not change through time.

#### Causation

Two additional tests were performed to address issues of reverse causation. On the one hand, we wanted to rule out bias from participants who had deceased shortly after an assessment, as they might have been ill and in increased need of social support prior to the assessment. We therefore re-ran the analyses, first, excluding 42 participants who had deceased within three months (90 days) after their last assessment, and second, excluding ninety participants who had deceased within one year (365 days) after their last assessment. Both re-analyses yielded results similar to our previous results. Since there are no substantial changes, we conclude that our findings are robust and do not contain such bias. On the other hand, we carried out an analysis with lagged variables, using predictor variables at a prior time point of observation *t*-1 and adjustment variables at time point *t* to model mortality risk at time point *t*. As the lagged analyses solely included respondents with two or more time points of observations, the sample was limited to 7,292 observations from 2,193 respondents. In this analysis, the fully adjusted hazard ratios for both network size and diversity turned insignificant, indicating that the predictive power of these network characteristics becomes weaker once the modelled time span to mortality outcomes is increased.

## Discussion

Our findings imply lowest risk of mortality for older adults who are embedded in personal networks that cover a large and heterogeneous set of social contacts. This is in line with earlier studies showing that structural characteristics (size and diversity) of the personal network are more strongly associated with a reduction in death hazard than functional characteristics [2, 5, 16]. Not only does the impact vary notably between the various characteristics of the personal network, but so do their pathways. Structural characteristics improve survival chances independently of mental, cognitive and physical conditions of an individual. In contrast, although there is no main association with functional characteristics, the perceived quality of social relationships potentially reduces mortality risk indirectly via other mechanisms, such as improved mental health. Our findings have several implications for current research on social relationships and survival.

First, it is particularly noteworthy that we did not find an association between emotional support and mortality risk. This finding puts into perspective arguments stating that older adults selectively choose with whom to affiliate, and invest only in relationships that entail emotionally supportive resources [19]. According to our results, successfully aging adults are able to maintain a resourceful structure and functionality in their network also when its composition changes (e.g. lost contacts are replaced by new contacts). Another explanation as to why functional aspects failed to directly relate to mortality in our study is that the measurement assessed received rather than perceived availability of support. The perception that a social contact would provide help when needed (often without actually calling on it) has been linked to positive health outcomes [13]. In contrast, receipt of support has been argued to adversely affect outcomes when it poses a threat to the recipient’s self-esteem or acts as an indirect marker of distress, as support is often provided only in response to stressful situations [27].

Second, we found only minor differences in associations over time, and between men and women, suggesting that rich networks yield life-long virtues for both the male and female population of older adults. The relative stability of associations over time may be explained by a delay in the effect of social integration on mortality. Supposedly, poor or deficient personal networks do not add immediately but only slowly to risk of mortality, e.g. through accumulated stress responses. However, a lagged analysis failed to confirm this delay argument in our data. Like in earlier research [28], living alone was more often negatively associated with mortality in men than in women in the age-adjusted model. Perhaps men are less able to compensate for deficits and less successfully call on support alternatives that temper the detrimental health impacts of social isolation [29].

Before concluding, three limitations of our study deserve attention. First, we used relatively simple measures of personal network structures, because the LASA data do not contain information about the interconnections between a respondent’s contacts (i.e. density). Also measures on alternative functional support types, such as providing advice, financial assistance or other tangible resources, would have been desirable. Second, whereas we treated functional and structural characteristics as theoretically distinct categories, they overlap, interact and reinforce one another in real life. For instance, large networks potentially pool a diverse set of resources high in support quality. Future research may also inquire, for example, whether some of the potentially detrimental effects of loneliness on health and survival are buffered by integration into certain network structures. Third, our study design did not allow to fully exclude reverse causality in the relation between network characteristics and health: declining personal network size and variation may activate deterioration of physical and cognitive functioning and *vice versa*, progressing impairments may hamper mobility and maintenance of social activities [30]. Cognitive and mental disorders even trigger social withdrawal in some older adults. This aggravates social isolation, which again reinforces ongoing declines in health, and so on. Finally, there was no information on negative social interactions, which, if they cause interpersonal strain [10] and stimulate unhealthy lifestyles [31], increase risk for disease.

Overall, our study highlights the benefits of a rigorous investigation of both functional and structural network characteristics, and the use of an appropriate follow-up design. Through using data from the Longitudinal Aging Study Amsterdam, we could follow networks of older adults repeatedly for two decades and hence closely relate personal network characteristics to the timing of mortality outcomes. Social integration has many facets, and these facets differ in their impact on longevity. If large and diverse personal networks indeed have the positive effects on survival as our findings suggest, then both future research and policy making might benefit from more insight into the conditions under which older adults succeed or fail in building and maintaining such personal networks.

## Supporting Information

S1 File Tables I to XXVII. Complete set of Cox proportional hazard regression models for the total population, and for men and women separately.
(RTF)
