# Supplementary material for: Personal Networks and Mortality Risk in Older Adults: A Twenty-Year Longitudinal Study
Source: PLoS One. 2015 Mar 3;10(3):e0116731. doi: 10.1371/journal.pone.0116731 (PMC4348168; doi:10.1371/journal.pone.0116731)
Supplement: S1 File — Complete set of Cox proportional hazard regression models for the total population, and for men and women separately. (RTF) [file pone.0116731.s001.rtf]

File S1: Supporting Information with Tables I to XXVII
Personal networks and mortality risk in older adults: A twenty-year longitudinal study

This document contains supporting information pertaining to the study on personal networks and mortality risk in older adults. The document is organized into three sections. It first shows the results for the total study population (i.e. all participants) and, after that, the results for the group of men and women, respectively. 
The first table in each section presents the extended Model 2, which included the total set of predictor variables, that is emotional loneliness, social loneliness, emotional support, instrumental support, living alone, contact frequency, network size and network diversity. Next, for every of the predictors, a table with the remaining models is provided: Model 1 tested the age-adjusted effect of a predictor. Model 3 adjusted for depression and anxiety (mental health). Model 4 adjusted for the MMSE-index (cognitive health). Model 5 adjusted for number of chronic diseases and ADL (physical health). Model 6 adjusted for all mental, cognitive and physical health variables.

I  Cox proportional hazard models for all participants
Table I		Extended model: Model 2
Table II	Emotional loneliness: Model 1 and Model 3-6 
Table III	Social loneliness: Model 1 and Model 3-6 
Table IV	Emotional support: Model 1 and Model 3-6 
Table V	Instrumental support: Model 1 and Model 3-6 
Table VI	Living alone: Model 1 and Model 3-6 
Table VII	Contact frequency: Model 1 and Model 3-6 
Table VIII	Network size: Model 1 and Model 3-6 
Table IX	Network diversity: Model 1 and Model 3-6 
II  Cox proportional hazard models for female participants
Table X	Extended model: Model 2 for female participants
Table XI	Emotional loneliness: Model 1 and Model 3-6 for female participants
Table XII	Social loneliness: Model 1 and Model 3-6 for female participants
Table XIII	Emotional support: Model 1 and Model 3-6 for female participants
Table XIV	Instrumental support: Model 1 and Model 3-6 for female participants
Table XV	living alone: Model 1 and Model 3-6 for female participants
Table XVI 	Contact frequency: Model 1 and Model 3-6 for female participants
Table XVII	Network size: Model 1 and Model 3-6 for female participants
Table XVIII	Network diversity: Model 1 and Model 3-6 for female participants
III  Cox proportional hazard models for male participants
Table XIX	Extended model: Model 2 for male participants
Table XX	Emotional loneliness: Model 1 and Model 3-6 for male participants
Table XXI	Social loneliness: Model 1 and Model 3-6 for male participants
Table XXII	Emotional support: Model 1 and Model 3-6 for male participants
Table XXIII	Instrumental support: Model 1 and Model 3-6 for male participants
Table XXIV	Living alone: Model 1 and Model 3-6 for male participants
Table XV	Contact frequency: Model 1 and Model 3-6 for male participants
Table XVI	Network size: Model 1 and Model 3-6 for male participants
Table XVII	Network diversity: Model 1 and Model 3-6 for male participants
I  Cox proportional hazard models for all participants

Table I
Extended model: Model 2
Death hazard ratios from Cox proportional hazard models for extended model with all predictors (Nind=2,911, Nobs=10,031)
	Model with age	Extended Model 2	
	HR (95% CI)	HR (95% CI)	
Age at baseline	1.108***	1.096***	
	(1.100,1.117)	(1.087,1.105)	
			
Emotional loneliness		1.053**	
		(1.017,1.090)	
			
Social loneliness		1.016	
		(0.973,1.062)	
			
Emotional support		0.975	
		(0.904,1.051)	
			
Instrumental support		1.154***	
		(1.063,1.252)	
			
Living alone		1.066	
		(0.929,1.223)	
			
Contact frequency		1.039	
		(0.969,1.113)	
			
Network size		0.988*	
		(0.977,0.999)	
			
Network diversity		0.957	
		(0.913,1.003)	
Notes. 95% confidence intervals in brackets. *p < 0.05, **p < 0.01, ***p < 0.001. All models were stratified by gender.
Table II
Emotional loneliness: Model 1 and Model 3-6 

Death hazard ratios from Cox proportional hazard models with emotional loneliness (Nind=2,911, Nobs=10,031)
	Model 1	Model 3	Model 4	Model 5	Model 6	
	HR (95% CI)	HR (95% CI)	HR (95% CI)	HR (95% CI)	HR (95% CI)	
Age at baseline	1.104***	1.101***	1.093***	1.081***	1.075***	
	(1.095,1.112)	(1.092,1.109)	(1.084,1.102)	(1.072,1.091)	(1.066,1.085)	
						
Emotional 	1.079***	1.020	1.073***	1.039*	1.023	
loneliness	(1.047,1.113)	(0.985,1.055)	(1.041,1.106)	(1.007,1.071)	(0.989,1.059)	
						
Depression		1.040***			1.015**	
		(1.029,1.051)			(1.004,1.026)	
						
Anxiety		0.968**			0.978	
		(0.944,0.991)			(0.955,1.002)	
						
Cognitive 			0.936***		0.952***	
functioning			(0.921,0.951)		(0.936,0.968)	
						
No. of chronic 				1.201***	1.205***	
diseases				(1.143,1.262)	(1.146,1.267)	
						
ADL				0.938***	0.946***	
				(0.928,0.948)	(0.936,0.957)	
Notes. 95% confidence intervals in brackets. *p < 0.05, **p < 0.01, ***p < 0.001. All models were stratified by gender.

Table III
Social loneliness: Model 1 and Model 3-6 

Death hazard ratios from Cox proportional hazard models with social loneliness (Nind=2,911, Nobs=10,031)
	Model 1	Model 3	Model 4	Model 5	Model 6	
	HR (95% CI)	HR (95% CI)	HR (95% CI)	HR (95% CI)	HR (95% CI)	
Age at baseline	1.106***	1.101***	1.096***	1.082***	1.076***	
	(1.098,1.115)	(1.092,1.110)	(1.087,1.105)	(1.073,1.091)	(1.067,1.085)	
						
Social loneliness	1.067***	1.028	1.060**	1.046*	1.030	
	(1.028,1.108)	(0.989,1.068)	(1.022,1.101)	(1.008,1.087)	(0.991,1.070)	
						
Depression		1.041***			1.016**	
		(1.030,1.051)			(1.005,1.026)	
						
Anxiety		0.968**			0.979	
		(0.945,0.991)			(0.956,1.002)	
						
Cognitive 			0.936***		0.953***	
functioning			(0.921,0.951)		(0.937,0.969)	
						
No. of chronic 				1.202***	1.205***	
diseases				(1.144,1.263)	(1.146,1.266)	
						
ADL				0.937***	0.946***	
				(0.927,0.947)	(0.936,0.957)	
Notes. 95% confidence intervals in brackets. *p < 0.05, **p < 0.01, ***p < 0.001. All models were stratified by gender.

Table IV
Emotional support: Model 1 and Model 3-6 

Death hazard ratios from Cox proportional hazard models with emotional support (Nind=2,911, Nobs=10,031)
	Model 1	Model 3	Model 4	Model 5	Model 6	
	HR (95% CI)	HR (95% CI)	HR (95% CI)	HR (95% CI)	HR (95% CI)	
Age at baseline	1.108***	1.101***	1.097***	1.083***	1.076***	
	(1.100,1.117)	(1.093,1.110)	(1.088,1.106)	(1.074,1.092)	(1.067,1.086)	
						
Emotional support	0.985	0.989	1.003	0.994	1.009	
	(0.917,1.058)	(0.921,1.062)	(0.934,1.077)	(0.926,1.067)	(0.940,1.083)	
						
Depression		1.042***			1.017**	
		(1.032,1.052)			(1.006,1.027)	
						
Anxiety		0.968**			0.979	
		(0.945,0.992)			(0.956,1.003)	
						
Cognitive 			0.934***		0.952***	
functioning			(0.919,0.949)		(0.936,0.968)	
						
No. of chronic 				1.201***	1.203***	
diseases				(1.143,1.263)	(1.144,1.264)	
						
ADL				0.936***	0.946***	
				(0.926,0.946)	(0.936,0.957)	
Notes. 95% confidence intervals in brackets. *p < 0.05, **p < 0.01, ***p < 0.001. All models were stratified by gender.


Table V
Instrumental support: Model 1 and Model 3-6 

Death hazard ratios from Cox proportional hazard models with instrumental support (Nind=2,911, Nobs=10,031)
	Model 1	Model 3	Model 4	Model 5	Model 6	
	HR (95% CI)	HR (95% CI)	HR (95% CI)	HR (95% CI)	HR (95% CI)	
Age at baseline	1.106***	1.100***	1.096***	1.083***	1.076***	
	(1.098,1.115)	(1.092,1.109)	(1.087,1.105)	(1.074,1.092)	(1.067,1.085)	
						
Instrumental 	1.137**	1.102*	1.127**	1.056	1.049	
support	(1.053,1.227)	(1.021,1.190)	(1.045,1.216)	(0.978,1.141)	(0.971,1.132)	
						
Depression		1.041***			1.017**	
		(1.031,1.051)			(1.006,1.027)	
						
Anxiety		0.968**			0.979	
		(0.945,0.992)			(0.956,1.003)	
						
Cognitive 			0.935***		0.952***	
functioning			(0.920,0.950)		(0.936,0.968)	
						
No. of chronic 				1.202***	1.203***	
diseases				(1.144,1.263)	(1.145,1.265)	
						
ADL				0.937***	0.947***	
				(0.927,0.947)	(0.936,0.958)	
Notes. 95% confidence intervals in brackets. *p < 0.05, **p < 0.01, ***p < 0.001. All models were stratified by gender.


Table VI
Living alone: Model 1 and Model 3-6 

Death hazard ratios from Cox proportional hazard models with living alone (Nind=2,911, Nobs=10,031)
	Model 1	Model 3	Model 4	Model 5	Model 6	
	HR (95% CI)	HR (95% CI)	HR (95% CI)	HR (95% CI)	HR (95% CI)	
Age at baseline	1.103***	1.099***	1.093***	1.081***	1.076***	
	(1.094,1.112)	(1.090,1.108)	(1.084,1.102)	(1.072,1.090)	(1.066,1.085)	
						
Living alone	1.230**	1.107	1.203**	1.097	1.051	
	(1.082,1.399)	(0.971,1.262)	(1.058,1.368)	(0.963,1.251)	(0.920,1.199)	
						
Depression		1.040***			1.016**	
		(1.030,1.050)			(1.006,1.027)	
						
Anxiety		0.971*			0.980	
		(0.947,0.995)			(0.957,1.004)	
						
Cognitive 			0.935***		0.952***	
functioning			(0.920,0.950)		(0.936,0.968)	
						
No. of chronic 				1.202***	1.203***	
diseases				(1.144,1.263)	(1.144,1.265)	
						
ADL				0.937***	0.947***	
				(0.927,0.947)	(0.936,0.958)	
Notes. 95% confidence intervals in brackets. *p < 0.05, **p < 0.01, ***p < 0.001. All models were stratified by gender.

Table VII
Contact frequency: Model 1 and Model 3-6 

Death hazard ratios from Cox proportional hazard models with contact frequency (Nind=2,911, Nobs=10,031)
	Model 1	Model 3	Model 4	Model 5	Model 6	
	HR (95% CI)	HR (95% CI)	HR (95% CI)	HR (95% CI)	HR (95% CI)	
Age at baseline	1.109***	1.102***	1.098***	1.084***	1.077***	
	(1.100,1.118)	(1.094,1.111)	(1.089,1.107)	(1.075,1.093)	(1.068,1.086)	
						
Contact 	1.075*	1.077*	1.052	1.060	1.048	
frequency	(1.012,1.143)	(1.014,1.143)	(0.991,1.117)	(0.998,1.126)	(0.988,1.112)	
						
Depression		1.042***			1.017**	
		(1.032,1.052)			(1.007,1.028)	
						
Anxiety		0.967**			0.978	
		(0.944,0.991)			(0.955,1.002)	
						
Cognitive 			0.935***		0.953***	
functioning			(0.921,0.951)		(0.937,0.969)	
						
No. of chronic 				1.199***	1.201***	
diseases				(1.141,1.260)	(1.143,1.263)	
						
ADL				0.936***	0.946***	
				(0.926,0.946)	(0.935,0.957)	
Notes. 95% confidence intervals in brackets. *p < 0.05, **p < 0.01, ***p < 0.001. All models were stratified by gender.


Table VIII
Network size: Model 1 and Model 3-6 

Death hazard ratios from Cox proportional hazard models with network size (Nind=2,911, Nobs=10,031)
	Model 1	Model 3	Model 4	Model 5	Model 6	
	HR (95% CI)	HR (95% CI)	HR (95% CI)	HR (95% CI)	HR (95% CI)	
Age at baseline	1.103***	1.098***	1.094***	1.080***	1.075***	
	(1.095,1.112)	(1.089,1.107)	(1.085,1.103)	(1.071,1.089)	(1.066,1.084)	
						
Network size	0.978***	0.982***	0.983***	0.982***	0.986***	
	(0.970,0.986)	(0.975,0.990)	(0.975,0.990)	(0.974,0.990)	(0.979,0.994)	
						
Depression		1.039***			1.015**	
		(1.029,1.049)			(1.004,1.026)	
						
Anxiety		0.970*			0.980	
		(0.947,0.993)			(0.957,1.003)	
						
Cognitive 			0.941***		0.957***	
functioning			(0.926,0.956)		(0.941,0.973)	
						
No. of chronic 				1.203***	1.205***	
diseases				(1.145,1.264)	(1.147,1.267)	
						
ADL				0.938***	0.947***	
				(0.928,0.948)	(0.936,0.958)	
Notes. 95% confidence intervals in brackets. *p < 0.05, **p < 0.01, ***p < 0.001. All models were stratified by gender.


Table IX
Network diversity: Model 1 and Model 3-6 

Death hazard ratios from Cox proportional hazard models with network diversity (Nind=2,911, Nobs=10,031)
	Model 1	Model 3	Model 4	Model 5	Model 6	
	HR (95% CI)	HR (95% CI)	HR (95% CI)	HR (95% CI)	HR (95% CI)	
Age at baseline	1.102***	1.097***	1.093***	1.078***	1.074***	
	(1.093,1.110)	(1.088,1.106)	(1.083,1.102)	(1.069,1.088)	(1.064,1.083)	
						
Network diversity	0.919***	0.937***	0.934***	0.932***	0.948**	
	(0.889,0.950)	(0.906,0.968)	(0.904,0.966)	(0.901,0.963)	(0.917,0.981)	
						
Depression		1.039***			1.015**	
		(1.029,1.049)			(1.004,1.026)	
						
Anxiety		0.971*			0.982	
		(0.948,0.995)			(0.959,1.005)	
						
Cognitive 			0.939***		0.955***	
functioning			(0.924,0.954)		(0.939,0.971)	
						
No. of chronic 				1.206***	1.207***	
diseases				(1.148,1.267)	(1.148,1.268)	
						
ADL				0.938***	0.947***	
				(0.928,0.948)	(0.936,0.958)	
Notes. 95% confidence intervals in brackets. *p < 0.05, **p < 0.01, ***p < 0.001. All models were stratified by gender.


I  Cox proportional hazard models for female participants

Table X
Extended model: Model 2 for female participants
Death hazard ratios from Cox proportional hazard models for extended model with all predictors (Nind=1,498, Nobs=5,391)
	Model with age	Extended Model 2	
	HR (95% CI)	HR (95% CI)	
Age at baseline	1.120***	1.106***	
	(1.107,1.134)	(1.091,1.121)	
			
Emotional loneliness		1.046	
		(0.994,1.100)	
			
Social loneliness		1.006	
		(0.937,1.079)	
			
Emotional support		0.863*	
		(0.764,0.976)	
			
Instrumental support		1.230**	
		(1.080,1.401)	
			
Living alone		1.046	
		(0.831,1.316)	
			
Contact frequency		0.963	
		(0.855,1.086)	
			
Network size		0.985	
		(0.968,1.003)	
			
Network diversity		0.979	
		(0.910,1.053)	
Notes. 95% confidence intervals in brackets. *p < 0.05, **p < 0.01, ***p < 0.001. 
Table XI
Emotional loneliness: Model 1 and Model 3-6 for female participants

Death hazard ratios from Cox proportional hazard models with emotional loneliness (Nind=1,498, Nobs=5,391)
	Model 1	Model 3	Model 4	Model 5	Model 6	
	HR (95% CI)	HR (95% CI)	HR (95% CI)	HR (95% CI)	HR (95% CI)	
Age at baseline	1.117***	1.112***	1.103***	1.090***	1.080***	
	(1.104,1.131)	(1.098,1.126)	(1.088,1.117)	(1.076,1.105)	(1.065,1.095)	
						
Emotional loneliness	1.064**	1.010	1.055*	1.034	1.018	
	(1.018,1.112)	(0.960,1.063)	(1.009,1.103)	(0.989,1.082)	(0.967,1.071)	
						
Depression		1.040***			1.017*	
		(1.024,1.055)			(1.001,1.033)	
						
Anxiety		0.956*			0.966	
		(0.923,0.990)			(0.933,1.001)	
						
Cognitive 			0.927***		0.939***	
functioning			(0.906,0.949)		(0.917,0.962)	
						
No. of chronic 				1.201***	1.200***	
diseases				(1.112,1.297)	(1.111,1.297)	
						
ADL				0.943***	0.951***	
				(0.928,0.958)	(0.935,0.967)	
Notes. 95% confidence intervals in brackets. *p < 0.05, **p < 0.01, ***p < 0.001. 

Table XII
Social loneliness: Model 1 and Model 3-6 for female participants

Death hazard ratios from Cox proportional hazard models with social loneliness (Nind=1,498, Nobs=5,391)
	Model 1	Model 3	Model 4	Model 5	Model 6	
	HR (95% CI)	HR (95% CI)	HR (95% CI)	HR (95% CI)	HR (95% CI)	
Age at baseline	1.119***	1.112***	1.103***	1.091***	1.080***	
	(1.105,1.132)	(1.098,1.126)	(1.089,1.118)	(1.076,1.106)	(1.065,1.095)	
						
Social loneliness	1.068*	1.029	1.062*	1.049	1.035	
	(1.008,1.131)	(0.969,1.092)	(1.003,1.125)	(0.990,1.111)	(0.975,1.099)	
						
Depression		1.039***			1.017*	
		(1.025,1.054)			(1.002,1.033)	
						
Anxiety		0.956*			0.966	
		(0.923,0.990)			(0.933,1.001)	
						
Cognitive 			0.926***		0.939***	
functioning			(0.905,0.948)		(0.917,0.962)	
						
No. of chronic 				1.202***	1.201***	
diseases				(1.113,1.297)	(1.112,1.297)	
						
ADL				0.942***	0.951***	
				(0.927,0.958)	(0.935,0.967)	
Notes. 95% confidence intervals in brackets. *p < 0.05, **p < 0.01, ***p < 0.001. 

Table XIII
Emotional support: Model 1 and Model 3-6 for female participants

Death hazard ratios from Cox proportional hazard models with emotional support (Nind=1,498, Nobs=5,391)
	Model 1	Model 3	Model 4	Model 5	Model 6	
	HR (95% CI)	HR (95% CI)	HR (95% CI)	HR (95% CI)	HR (95% CI)	
Age at baseline	1.119***	1.111***	1.104***	1.090***	1.080***	
	(1.105,1.133)	(1.098,1.125)	(1.090,1.119)	(1.076,1.105)	(1.065,1.095)	
						
Emotional support	0.884*	0.882*	0.916	0.889*	0.920	
	(0.788,0.992)	(0.786,0.991)	(0.817,1.027)	(0.793,0.997)	(0.820,1.032)	
						
Depression		1.040***			1.018*	
		(1.026,1.055)			(1.003,1.034)	
						
Anxiety		0.959*			0.968	
		(0.926,0.994)			(0.935,1.003)	
						
Cognitive 			0.927***		0.941***	
functioning			(0.906,0.949)		(0.918,0.964)	
						
No. of chronic 				1.205***	1.202***	
diseases				(1.116,1.301)	(1.112,1.298)	
						
ADL				0.942***	0.952***	
				(0.927,0.958)	(0.936,0.968)	
Notes. 95% confidence intervals in brackets. *p < 0.05, **p < 0.01, ***p < 0.001. 


Table XIV
Instrumental support: Model 1 and Model 3-6 for female participants

Death hazard ratios from Cox proportional hazard models with instrumental support (Nind=1,498, Nobs=5,391)
	Model 1	Model 3	Model 4	Model 5	Model 6	
	HR (95% CI)	HR (95% CI)	HR (95% CI)	HR (95% CI)	HR (95% CI)	
Age at baseline	1.118***	1.111***	1.103***	1.091***	1.080***	
	(1.105,1.132)	(1.097,1.125)	(1.089,1.117)	(1.076,1.106)	(1.065,1.095)	
						
Instrumental 	1.149*	1.118	1.147*	1.053	1.053	
support	(1.019,1.296)	(0.991,1.261)	(1.018,1.293)	(0.933,1.189)	(0.933,1.187)	
						
Depression		1.040***			1.018*	
		(1.025,1.054)			(1.003,1.034)	
						
Anxiety		0.957*			0.967	
		(0.924,0.991)			(0.933,1.001)	
						
Cognitive 			0.926***		0.939***	
functioning			(0.904,0.947)		(0.917,0.962)	
						
No. of chronic 				1.200***	1.197***	
diseases				(1.111,1.296)	(1.108,1.293)	
						
ADL				0.942***	0.952***	
				(0.927,0.958)	(0.936,0.968)	
Notes. 95% confidence intervals in brackets. *p < 0.05, **p < 0.01, ***p < 0.001. 


Table XV
Living alone: Model 1 and Model 3-6 for female participants

Death hazard ratios from Cox proportional hazard models with living alone (Nind=1,498, Nobs=5,391)
	Model 1	Model 3	Model 4	Model 5	Model 6	
	HR (95% CI)	HR (95% CI)	HR (95% CI)	HR (95% CI)	HR (95% CI)	
Age at baseline	1.116***	1.110***	1.100***	1.090***	1.080***	
	(1.101,1.130)	(1.095,1.125)	(1.085,1.116)	(1.074,1.106)	(1.064,1.096)	
						
Living alone	1.184	1.101	1.176	1.060	1.030	
	(0.950,1.476)	(0.883,1.374)	(0.943,1.467)	(0.849,1.323)	(0.825,1.288)	
						
Depression		1.040***			1.018*	
		(1.025,1.055)			(1.003,1.034)	
						
Anxiety		0.958*			0.967	
		(0.925,0.992)			(0.934,1.002)	
						
Cognitive 			0.926***		0.939***	
functioning			(0.904,0.947)		(0.917,0.962)	
						
No. of chronic 				1.200***	1.198***	
diseases				(1.111,1.296)	(1.109,1.294)	
						
ADL				0.942***	0.951***	
				(0.927,0.957)	(0.935,0.967)	
Notes. 95% confidence intervals in brackets. *p < 0.05, **p < 0.01, ***p < 0.001. 

Table XVI
Contact frequency: Model 1 and Model 3-6 for female participants

Death hazard ratios from Cox proportional hazard models with contact frequency (Nind=1,498, Nobs=5,391)
	Model 1	Model 3	Model 4	Model 5	Model 6	
	HR (95% CI)	HR (95% CI)	HR (95% CI)	HR (95% CI)	HR (95% CI)	
Age at baseline	1.120***	1.113***	1.105***	1.091***	1.080***	
	(1.107,1.134)	(1.099,1.127)	(1.090,1.119)	(1.077,1.106)	(1.065,1.095)	
						
Contact frequency	0.997	1.009	0.984	0.995	0.986	
	(0.902,1.101)	(0.914,1.114)	(0.893,1.084)	(0.901,1.099)	(0.895,1.087)	
						
Depression		1.041***			1.018*	
		(1.026,1.055)			(1.003,1.034)	
						
Anxiety		0.957*			0.967	
		(0.924,0.991)			(0.934,1.001)	
						
Cognitive 			0.925***		0.939***	
functioning			(0.904,0.947)		(0.917,0.962)	
						
No. of chronic 				1.201***	1.199***	
diseases				(1.112,1.298)	(1.110,1.296)	
						
ADL				0.942***	0.951***	
				(0.926,0.957)	(0.935,0.967)	
Notes. 95% confidence intervals in brackets. *p < 0.05, **p < 0.01, ***p < 0.001. 


Table XVII
Network size: Model 1 and Model 3-6 for female participants

Death hazard ratios from Cox proportional hazard models with network size (Nind=1,498, Nobs=5,391)
	Model 1	Model 3	Model 4	Model 5	Model 6	
	HR (95% CI)	HR (95% CI)	HR (95% CI)	HR (95% CI)	HR (95% CI)	
Age at baseline	1.116***	1.109***	1.102***	1.088***	1.079***	
	(1.102,1.129)	(1.095,1.123)	(1.088,1.117)	(1.073,1.103)	(1.064,1.094)	
						
Network size	0.982**	0.986*	0.988	0.983**	0.989	
	(0.970,0.994)	(0.974,0.998)	(0.976,1.000)	(0.971,0.995)	(0.977,1.002)	
						
Depression		1.039***			1.017*	
		(1.024,1.054)			(1.002,1.032)	
						
Anxiety		0.957*			0.967	
		(0.925,0.991)			(0.934,1.002)	
						
Cognitive 			0.929***		0.943***	
functioning			(0.908,0.952)		(0.920,0.966)	
						
No. of chronic 				1.207***	1.203***	
diseases				(1.117,1.303)	(1.114,1.300)	
						
ADL				0.943***	0.951***	
				(0.928,0.958)	(0.935,0.967)	
Notes. 95% confidence intervals in brackets. *p < 0.05, **p < 0.01, ***p < 0.001. 


Table XVIII
Network diversity: Model 1 and Model 3-6 for female participants

Death hazard ratios from Cox proportional hazard models with network diversity (Nind=1,498, Nobs=5,391)
	Model 1	Model 3	Model 4	Model 5	Model 6	
	HR (95% CI)	HR (95% CI)	HR (95% CI)	HR (95% CI)	HR (95% CI)	
Age at baseline	1.113***	1.107***	1.100***	1.086***	1.077***	
	(1.099,1.128)	(1.093,1.122)	(1.086,1.115)	(1.071,1.101)	(1.062,1.093)	
						
Network diversity	0.928**	0.942*	0.946*	0.932**	0.953	
	(0.881,0.979)	(0.894,0.993)	(0.897,0.997)	(0.885,0.983)	(0.904,1.005)	
						
Depression		1.039***			1.017*	
		(1.025,1.054)			(1.002,1.033)	
						
Anxiety		0.959*			0.968	
		(0.926,0.993)			(0.935,1.002)	
						
Cognitive 			0.928***		0.942***	
functioning			(0.907,0.950)		(0.919,0.965)	
						
No. of chronic 				1.207***	1.204***	
diseases				(1.118,1.304)	(1.114,1.300)	
						
ADL				0.943***	0.951***	
				(0.928,0.958)	(0.936,0.967)	
Notes. 95% confidence intervals in brackets. *p < 0.05, **p < 0.01, ***p < 0.001. 


I  Cox proportional hazard models for male participants

Table XIX
Extended model: Model 2 for male participants
Death hazard ratios from Cox proportional hazard models for extended model with all predictors (Nind=1,413, Nobs=4,460)
	Model with age	Extended Model 2	
	HR (95% CI)	HR (95% CI)	
Age at baseline	1.100***	1.089***	
	(1.089,1.111)	(1.077,1.100)	
			
Emotional loneliness		1.064*	
		(1.013,1.116)	
			
Social loneliness		1.020	
		(0.964,1.079)	
			
Emotional support		1.065	
		(0.967,1.172)	
			
Instrumental support		1.099	
		(0.989,1.222)	
			
Living alone		1.053	
		(0.882,1.257)	
			
Contact frequency		1.085	
		(0.995,1.184)	
			
Network size		0.990	
		(0.976,1.004)	
			
Network diversity		0.945	
		(0.889,1.005)	
Notes. 95% confidence intervals in brackets. *p < 0.05, **p < 0.01, ***p < 0.001. 
Table XX
Emotional loneliness: Model 1 and Model 3-6 for male participants

Death hazard ratios from Cox proportional hazard models with emotional loneliness (Nind=1,413, Nobs=4,460)
	Model 1	Model 3	Model 4	Model 5	Model 6	
	HR (95% CI)	HR (95% CI)	HR (95% CI)	HR (95% CI)	HR (95% CI)	
Age at baseline	1.094***	1.092***	1.087***	1.076***	1.072***	
	(1.083,1.105)	(1.081,1.104)	(1.075,1.098)	(1.064,1.087)	(1.061,1.084)	
						
Emotional 	1.096***	1.030	1.091***	1.044	1.027	
loneliness	(1.051,1.143)	(0.983,1.080)	(1.046,1.137)	(1.000,1.089)	(0.980,1.076)	
						
Depression		1.039***			1.014	
		(1.024,1.054)			(0.998,1.029)	
						
Anxiety		0.980			0.989	
		(0.948,1.014)			(0.958,1.022)	
						
Cognitive 			0.946***		0.963**	
functioning			(0.925,0.967)		(0.941,0.986)	
						
No. of chronic 				1.204***	1.206***	
diseases				(1.128,1.285)	(1.129,1.288)	
						
ADL				0.935***	0.943***	
				(0.921,0.948)	(0.928,0.958)	
Notes. 95% confidence intervals in brackets. *p < 0.05, **p < 0.01, ***p < 0.001. 

Table XXI
Social loneliness: Model 1 and Model 3-6 for male participants

Death hazard ratios from Cox proportional hazard models with social loneliness (Nind=1,413, Nobs=4,460)
	Model 1	Model 3	Model 4	Model 5	Model 6	
	HR (95% CI)	HR (95% CI)	HR (95% CI)	HR (95% CI)	HR (95% CI)	
Age at baseline	1.098***	1.093***	1.090***	1.077***	1.073***	
	(1.087,1.109)	(1.082,1.104)	(1.079,1.102)	(1.066,1.088)	(1.062,1.085)	
						
Social loneliness	1.067**	1.026	1.060*	1.045	1.027	
	(1.016,1.120)	(0.976,1.079)	(1.010,1.113)	(0.995,1.098)	(0.976,1.081)	
						
Depression		1.041***			1.015*	
		(1.027,1.056)			(1.000,1.030)	
						
Anxiety		0.981			0.990	
		(0.949,1.014)			(0.958,1.022)	
						
Cognitive 			0.945***		0.964**	
functioning			(0.925,0.966)		(0.942,0.987)	
						
No. of chronic 				1.205***	1.205***	
diseases				(1.129,1.286)	(1.129,1.287)	
						
ADL				0.933***	0.943***	
				(0.919,0.947)	(0.928,0.958)	
Notes. 95% confidence intervals in brackets. *p < 0.05, **p < 0.01, ***p < 0.001. 

Table XXII
Emotional support: Model 1 and Model 3-6 for male participants

Death hazard ratios from Cox proportional hazard models with emotional support (Nind=1,413, Nobs=4,460)
	Model 1	Model 3	Model 4	Model 5	Model 6	
	HR (95% CI)	HR (95% CI)	HR (95% CI)	HR (95% CI)	HR (95% CI)	
Age at baseline	1.100***	1.094***	1.091***	1.078***	1.073***	
	(1.089,1.111)	(1.083,1.105)	(1.080,1.103)	(1.066,1.089)	(1.062,1.085)	
						
Emotional support	1.059	1.067	1.068	1.067	1.074	
	(0.966,1.160)	(0.975,1.169)	(0.976,1.170)	(0.975,1.168)	(0.982,1.175)	
						
Depression		1.043***			1.016*	
		(1.029,1.057)			(1.002,1.031)	
						
Anxiety		0.981			0.989	
		(0.949,1.014)			(0.958,1.022)	
						
Cognitive 			0.943***		0.963**	
functioning			(0.922,0.964)		(0.941,0.986)	
						
No. of chronic 				1.205***	1.205***	
diseases				(1.129,1.286)	(1.128,1.286)	
						
ADL				0.932***	0.943***	
				(0.918,0.945)	(0.928,0.958)	
Notes. 95% confidence intervals in brackets. *p < 0.05, **p < 0.01, ***p < 0.001. 


Table XXIII
Instrumental support: Model 1 and Model 3-6 for male participants

Death hazard ratios from Cox proportional hazard models with instrumental support (Nind=1,413, Nobs=4,460)
	Model 1	Model 3	Model 4	Model 5	Model 6	
	HR (95% CI)	HR (95% CI)	HR (95% CI)	HR (95% CI)	HR (95% CI)	
Age at baseline	1.098***	1.093***	1.090***	1.078***	1.073***	
	(1.088,1.109)	(1.082,1.104)	(1.079,1.102)	(1.066,1.089)	(1.062,1.085)	
						
Instrumental 	1.126*	1.089	1.114*	1.059	1.048	
support	(1.020,1.244)	(0.986,1.203)	(1.010,1.230)	(0.958,1.171)	(0.949,1.158)	
						
Depression		1.041***			1.016*	
		(1.027,1.056)			(1.001,1.031)	
						
Anxiety		0.981			0.990	
		(0.949,1.014)			(0.958,1.022)	
						
Cognitive 			0.944***		0.964**	
functioning			(0.923,0.965)		(0.942,0.986)	
						
No. of chronic 				1.206***	1.206***	
diseases				(1.130,1.287)	(1.129,1.288)	
						
ADL				0.933***	0.943***	
				(0.919,0.947)	(0.929,0.958)	
Notes. 95% confidence intervals in brackets. *p < 0.05, **p < 0.01, ***p < 0.001. 


Table XXIV
Living alone: Model 1 and Model 3-6 for male participants

Death hazard ratios from Cox proportional hazard models with living alone (Nind=1,413, Nobs=4,460)
	Model 1	Model 3	Model 4	Model 5	Model 6	
	HR (95% CI)	HR (95% CI)	HR (95% CI)	HR (95% CI)	HR (95% CI)	
Age at baseline	1.095***	1.092***	1.088***	1.076***	1.073***	
	(1.084,1.107)	(1.081,1.104)	(1.076,1.100)	(1.065,1.088)	(1.061,1.085)	
						
Living alone	1.243**	1.094	1.212*	1.109	1.057	
	(1.060,1.459)	(0.927,1.292)	(1.032,1.422)	(0.942,1.305)	(0.895,1.248)	
						
Depression		1.040***			1.015*	
		(1.026,1.055)			(1.000,1.030)	
						
Anxiety		0.984			0.991	
		(0.952,1.018)			(0.959,1.024)	
						
Cognitive 			0.945***		0.964**	
functioning			(0.924,0.966)		(0.942,0.986)	
						
No. of chronic 				1.206***	1.205***	
diseases				(1.130,1.287)	(1.128,1.287)	
						
ADL				0.933***	0.943***	
				(0.920,0.947)	(0.928,0.958)	
Notes. 95% confidence intervals in brackets. *p < 0.05, **p < 0.01, ***p < 0.001. 

Table XXV
Contact frequency: Model 1 and Model 3-6 for male participants

Death hazard ratios from Cox proportional hazard models with contact frequency (Nind=1,413, Nobs=4,460)
	Model 1	Model 3	Model 4	Model 5	Model 6	
	HR (95% CI)	HR (95% CI)	HR (95% CI)	HR (95% CI)	HR (95% CI)	
Age at baseline	1.101***	1.095***	1.093***	1.079***	1.075***	
	(1.090,1.112)	(1.084,1.106)	(1.082,1.105)	(1.068,1.091)	(1.063,1.087)	
						
Contact frequency	1.124**	1.116**	1.099*	1.100*	1.088*	
	(1.041,1.214)	(1.035,1.204)	(1.018,1.186)	(1.019,1.187)	(1.009,1.174)	
						
Depression		1.043***			1.017*	
		(1.028,1.057)			(1.003,1.032)	
						
Anxiety		0.979			0.987	
		(0.947,1.012)			(0.955,1.020)	
						
Cognitive 			0.947***		0.966**	
functioning			(0.926,0.968)		(0.944,0.989)	
						
No. of chronic 				1.202***	1.202***	
diseases				(1.127,1.283)	(1.126,1.284)	
						
ADL				0.932***	0.943***	
				(0.919,0.946)	(0.928,0.958)	
Notes. 95% confidence intervals in brackets. *p < 0.05, **p < 0.01, ***p < 0.001. 


Table XVI
Network size: Model 1 and Model 3-6 for male participants

Death hazard ratios from Cox proportional hazard models with network size (Nind=1,413, Nobs=4,460)
	Model 1	Model 3	Model 4	Model 5	Model 6	
	HR (95% CI)	HR (95% CI)	HR (95% CI)	HR (95% CI)	HR (95% CI)	
Age at baseline	1.095***	1.091***	1.089***	1.075***	1.072***	
	(1.084,1.106)	(1.080,1.102)	(1.077,1.100)	(1.064,1.087)	(1.060,1.083)	
						
Network size	0.976***	0.981***	0.979***	0.981***	0.985**	
	(0.966,0.986)	(0.971,0.991)	(0.970,0.989)	(0.972,0.991)	(0.975,0.995)	
						
Depression		1.038***			1.014	
		(1.024,1.053)			(0.999,1.029)	
						
Anxiety		0.983			0.990	
		(0.951,1.016)			(0.958,1.023)	
						
Cognitive 			0.952***		0.968**	
functioning			(0.931,0.974)		(0.946,0.991)	
						
No. of chronic 				1.203***	1.204***	
diseases				(1.127,1.283)	(1.127,1.285)	
						
ADL				0.935***	0.944***	
				(0.921,0.949)	(0.929,0.959)	
Notes. 95% confidence intervals in brackets. *p < 0.05, **p < 0.01, ***p < 0.001. 


Table XXVII
Network diversity: Model 1 and Model 3-6 for male participants

Death hazard ratios from Cox proportional hazard models with network diversity (Nind=1,413, Nobs=4,460)
	Model 1	Model 3	Model 4	Model 5	Model 6	
	HR (95% CI)	HR (95% CI)	HR (95% CI)	HR (95% CI)	HR (95% CI)	
Age at baseline	1.094***	1.090***	1.087***	1.074***	1.071***	
	(1.083,1.105)	(1.079,1.101)	(1.076,1.099)	(1.063,1.086)	(1.059,1.083)	
						
Network diversity	0.916***	0.936**	0.928***	0.933**	0.947*	
	(0.877,0.956)	(0.897,0.978)	(0.889,0.969)	(0.894,0.974)	(0.906,0.989)	
						
Depression		1.039***			1.014	
		(1.024,1.053)			(0.999,1.029)	
						
Anxiety		0.985			0.993	
		(0.953,1.019)			(0.961,1.026)	
						
Cognitive 			0.949***		0.967**	
functioning			(0.928,0.971)		(0.944,0.989)	
						
No. of chronic 				1.207***	1.206***	
diseases				(1.131,1.288)	(1.129,1.288)	
						
ADL				0.934***	0.943***	
				(0.921,0.948)	(0.929,0.958)	
Notes. 95% confidence intervals in brackets. *p < 0.05, **p < 0.01, ***p < 0.001. 


 
